# Boutonneuse Fever in Southeastern Romania

**DOI:** 10.3390/microorganisms11112734

**Published:** 2023-11-09

**Authors:** Simona Claudia Cambrea, Diana Badiu, Constantin Ionescu, Roxana Penciu, Loredana Pazara, Cristina Maria Mihai, Mara Andreea Cambrea, Larisia Mihai

**Affiliations:** 1 Faculty of Medicine, “Ovidius” University from Constanta, 900470 Constanta, Romania; cambrea.claudia@gmail.com (S.C.C.); roxanapenciu@yahoo.com (R.P.); loredanapazara@yahoo.com (L.P.); cristina2603@yahoo.com (C.M.M.); cambrea.andreea@gmail.com (M.A.C.); larisia_mihai@yahoo.com (L.M.); 2 Clinical Hospital of Infectious Diseases, 900178 Constanta, Romania

**Keywords:** boutonneuse fever, Dobruja region, *Rickettsia conorii*, southeastern Romania

## Abstract

Boutonneuse fever (BF) is an eruptive disease and is classified as a spotted fever, which is endemic in the Mediterranean basin (i.e., Marseille fever or Mediterranean spotted fever) and the Black Sea, caused by *Rickettsia conorii*, with dog ticks being a vector (i.e., *Rhipicephalus sanguineus*). In Romania, although the first reported outbreak of BF occurred during the summer of 1931 in Constanta, the disease was discovered in 1910. Although the disease has occurred most frequently in the two counties of the Dobruja region (Constanta and Tulcea), a region of the Balkan Peninsula, during the last few years, other counties in southeastern Romania have started to report BF cases. In a period of 9 years, 533 cases were registered in Constanta county, while in a period of 11 years, 339 cases were registered in Bucharest county. In this review, we describe the bacterial tick-borne disease caused by *R. conorii* in southeastern Romania, focusing on its history and epidemiology, pathophysiology, clinical aspects, diagnosis, treatment and preventive measures in the context of climate changes. Although *R. conorii* is the principal etiologic agent of BF in southeastern Romania, we should take into consideration that other Rickettsia spp. could be present and involved in disease transmission.

## 1. Introduction

Boutonneuse, French for spotty fever (BF) or Mediterranean spotted fever (MSF), is a tick-borne disease caused by *Rickettsia conorii* and transmitted to humans by the brown dog tick, *Rhipicephalus sanguineus*. BF is traditionally considered to be endemic to the regions bordering the Mediterranean basin, including southern Europe and northern Africa. Among the European countries with relatively high *R. conorii* infection rates are Portugal, Spain, France and Italy [1].

The tick *R. sanguineus* is also the main reservoir of a pathogen from *R. conorii* due to rickettsial permanent transstadial perpetuation which ensures the permanent survival of bacteria [2]. The most important natural reservoirs for *R. conorii* in Romania are dogs, but other mammalian species may also be involved like sheep, cattle and, very rarely, cats [3]. It is well known that in tropical and subtropical areas, *R. sanguineus* is found throughout the year [4]. In temperate regions like Romania, this tick is found during late spring and early autumn [5]. 

In a study performed by Sandor and contributors, they found that in wetlands of the Danube Delta, *Rickettsia rossicus* had a dominant occurrence in dogs from this area [6]. 

Although the disease occurs most frequently in Constanta and Tulcea counties, which includes in its northeast corner the large and thinly populated estuary of the Danube, recently, other counties have started to report cases of BF: Prahova, Dîmbovita, Calarași and Buzău [7]. Throughout Central Europe, including Romania, isolated cases of BF have been reported [8]. In the western counties of Romania, since this condition is not endemic, the disease is more difficult to be recognized and diagnosed; but, with the complete anamnesis of patients including recent holidays to these endemic areas, the disease should be taken into consideration. 

We searched PubMed, EMBASE and Web of Science to find all articles published until August 2023. All English- and non-English-language articles were included. We considered both reviews and observational studies, conducted in general populations and risk groups. The search terms were as follows: BF in southeastern Romania, BF from Constanta and Tulcea counties, Rickettsia sp. involved in BF transmission and bacterial tick-borne disease from Dobruja region. The excluded articles were those with inconsistent information, an inappropriate study design or a lack of suitable testing. 

In this review, we describe the BF caused by *R. conorii*, a bacterial tick-borne disease in southeastern Romania, focusing on its history and epidemiology, pathophysiology, clinical aspects, diagnosis, treatment and preventive measures.

## 2. History and Epidemiology

The disease was first described in Tunis in 1920 by Connor and Bruch, and its name is related to a papular skin rash noticed during disease discovery [9]. Carducci in 1920 in Italy and Olmer in Marseilles in 1925 described a Mediterranean feverish disease that in 1932 received the name of Mediterranean BF. Meanwhile, in 1925, Pieri described the *tache noir*, or black spot or inoculation eschar, as characteristic of the disease. In 1930, Durand and Conseil showed the role of the dog tick *R. sanguineus* in disease transmission. In 1932, Brumpt discovered the causal agent, a rickettsia that he named in honor of Connor: *R. conorii* [10].

In Romania, the first reported outbreak of BF occurred during the summer of 1931 in Constanta county, involving 34 individuals. Suspicion of a new disease different to typhus had been diagnosed in Romania since 1910 (i.e., 11 persons in 1910 and 4 persons between 1932 and 1934) [11,12]. 

Its appearance in a non-Mediterranean country was most probably because of intense commerce with sheep between two harbors, Marseille in France and Constanta in Romania. At that moment, it was considered that sheep were the main reservoir of R. conorii [3,13]. In 1931, it was considered that the number of cases in Constanta was higher because there were more inapparent or oligosymptomatic cases [3,13]. After World War II, the disease spread in the Bucharest area, between 1948 and 1951, with 89 cases detected [12]. The isolation of rickettsia from the blood of one of the patients and the presence of dogs parasitized by *R. sanguineus* seems to appear between 1931 and 1948 [11,12].

The overall incidence of BF varied between 0.3 and 4.2 per 100,000 inhabitants in the period 2000–2016 in southeastern Romania. The highest incidence was observed in two counties in the Dobruja region: Tulcea county with an incidence of 15.76 per 100,000 inhabitants and Constanta county with an incidence of 5.73 per 100,000 inhabitants. In 2016, the highest number of cases was registered, especially in May and July [7]. 

**Figure 1:**
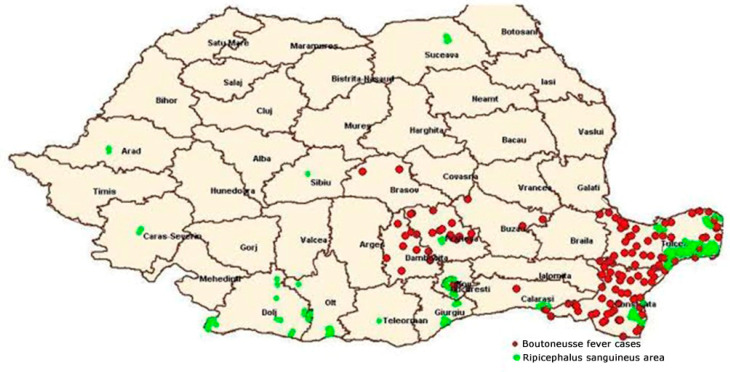
Distribution of BF in correlation with the spread area of *R. sanguineus*—Romania in 2014 (according to data from the National Institute of Public Health and National Center for Surveillance and Control of Infectious Diseases) [7].

BF still represents a public health problem in Constanta and Tulcea counties, as we can see in Figure 1 [7].

Recently, the study of Ivan and contributors [14] showed, for the first time, the discovery of *Rickettsia hoogstraalii* in Romania and also *R. rossicus* as ticks. In this study, five species of rickettsia were identified. This new species of *R. hoogstraalii* was described for the first time in Croatia in 2006, and its pathogenicity is currently not well known. Also, the detection of *Rickettsia raoultii* and *Rickettsia monacensis* in unfed *Haemaphysalis punctata* larvae supports the hypothesis of rickettsial transmission from female ticks to larvae. Considering this last aspect, the bite of larvae could present a transmission risk for this disease, which should be studied more in the future [14].

*R. conorii* is a Gram-negative, intracellular bacterium, which retains fuchsin by the Gimenez technique [15]. More recently, the species has been classified into four groups: a spotted fever group (i.e., *Rickettsia rickettsii*, *R. conorii* and others); a typhus group (i.e., *Rickettsia prowazekii* and *Rickettsia typhi*); an ancestral group; and the recently formed transitional group [16]. 

Even if it is a long or short journey, all age groups are at risk for rickettsial infections during visits to endemic areas. The transmission risk increases with time spent engaging in outdoor activities, especially during the lifecycle activity for the vector. In many parts of the world, however, rickettsial infections occur year-round. The commonly diagnosed rickettsial diseases in travelers are in the spotted fever groups [17].

Interestingly, genetic techniques have made it possible to dig much deeper into the rickettsial field by unraveling the rickettsial taxonomy [18]. Some authors showed that *R. conorii* strains could be divided into other subspecies like *Rickettsia conorii caspia*, *Rickettsia conorii israelensis*, *Rickettsia conorii indica* and *Rickettsia conorii conorii* [19]. 

After rickettsiae infect the host, they multiply in organs including fluids of the tick which transmit the disease by using rostra [20]. The vector of *R. conorii* is the dog tick *R. sanguineus*, first named by Durand and Conseil in 1930 [10]. It was shown that the ticks have a more important role in the rickettsia lifecycle [21]. 

Other species like *R. conorii israelensis* have been discovered in different areas including sub-Saharan Africa, India, Greece, Turkey, Bulgaria and Ukraine. Although all these species are different in genetic morphology, they present a similar clinical aspect [22].

## 3. Pathophysiology

The main target of *R. conorii* is the endothelial cells of small and medium blood vessels, but also macrophages and hepatocytes [23]. After the infection of human endothelial cells, vascular permeability increases, as well as inflammation, but also the infiltration of immune cells through mechanisms not yet fully clarified [24]. During rickettsial infection, several vasoactive mediators are produced by endothelial cells [25]. The transcriptional activation of cyclooxygenase-2 occurs, leading to robust prostaglandin secretion. In the context of rickettsioses, the damage of the endothelial cells is mediated by oxidants. This is also supported by the severe evolution of the disease in patients with glucose-6-phosphate dehydrogenase deficiency. During endothelial activation, two major signaling cascades, nuclear factor kB and mitogen-activated protein kinase, are activated to produce proinflammatory cytokines [25]. These cytokines increase the expression of cells’ adhesion molecules that allow the recruitment of leukocytes to the inflammation site [26].

The host’s response to *R. conorii* is the production of interferon beta (IFN-β) by infected endothelial cells. IFN-β causes the activation of the transducer and activator of transcription protein families, which subsequently interfere with rickettsial replication in host cells [27]. Mechanisms involved in the intracellular destruction of Rickettsia spp. are nitric oxide synthesis, hydrogen peroxide production and tryptophan degradation. Macrophages, natural killer (NK) cells and T lymphocytes produce IFN-γ and tumor necrosis factor alpha which act synergically to induce nitric oxide production in endothelial cells [28]. In human macrophages, the eradication of bacteria is achieved by the production of the enzyme indoleamine-pyrrole 2,3-dioxygenase, which, through degradation, limits the availability of tryptophan, leading to the starvation of the bacteria [14]. Dendritic cells (DCs) play an important role in the immune response against rickettsial infections by increasing CD4+, CD8+, NK and IFN-γ cell production [29].

Although much is known about the pathogenesis of this disease, many other aspects of *R. conorii* infection remain obscure [10].

## 4. Clinical Aspects

Rickettsia reach the human body at the level of skin, after a tick bite, and then spread in the body through the lymphatic vessels or circulatory system. Through the circulatory system, they reach the level of endothelial cells in the skin, producing button-like papules. At the place where a tick bites the skin, a lesion with the aspect of an eschar is formed, and its presence may suggest the disease. The eschar (black spot) is painless, sometimes necrotic or like a cigarette burn, and can be confused with scratch lesions or a boil. In addition to the rash and black spot, patients present fever, headache and myalgias [29,30]. BF has been observed to produce fever and maculopapular rash [31]. Fever is the most common in all cases at an interval of six to sixteen days after inoculation [31]. These symptoms were similar in different regions of the country, as we can see in Table 1. 

In children, the symptoms have been shown to be similar to those in adults, but in more mild forms [35]. Therefore, a higher incidence of gastrointestinal symptoms, lymphadenopathy, hepatosplenomegaly, arthralgia and myalgia were seen in children [36]. When treatment is first initiated, the symptoms seems to disappear after 48 h, with the disease being removed within approximately 10 days [31]. Although in some areas around the globe, it has been shown that fluroquinolone administration in adults is positively associated with increased disease severity [37], in our area, this aspect was not observed. 

Complications of BF have been also reported, like coronary ectasia and atrial fibrillation [38]; neurological manifestations [39]; renal failure [40]; intraocular inflammation, which sometimes is considered place of inoculation [41]; or pancreatitis [42]. These complications were not seen as often in children than in adults. The suspicion of the disease is of great importance in order to avoid delayed treatment [43]. The evolution of the disease is favorable in most cases, and mortality is low, at around 2–5% [44].

Another two aspects which could be observed in patients and can influence decreasing the number of cases is the prompt initiation of prophylaxis after a tick bite. In Constanta county, no deaths were recorded, and just a few cases had neurological manifestation [45]. 

In another study from Romania, between 2000 and 2001 performed in Bucharest were diagnosed over 339 patients with BF at the National Institute of Infectious Diseases “Prof. Dr. Matei Bals” [3]. The majority of them presented with fever (i.e., 99.4%). Other clinical symptoms noticed in this study were headache in 43.1%, myalgias in 43.4%, arthralgias in 27.8%, renal function impairment in 22.8%, respiratory symptoms in 16.8% and central nervous system impairment in 4.7% of patients. From a laboratory point of view, they noticed an increased level of leukocytes in 31.8% and a decreased level of thrombocytes in 50.9%. The serum fibrinogen and transaminase levels were increased in 55.1% and 79.5% of patients, respectively. Serology for *R. conorii* was positive in 36.28% of patients. In this study, there were no deaths recorded. The results of the study supported the fact that in Romania, BF should be considered in patients with fever even in the case of unrecognized tick exposure [34]. 

In Constanta county, between 2006 and 2015, we noticed a total of 533 cases of BF. From these cases, 97.5% presented fever, 80.4% presented headache, 80.3% presented myalgias and 97.18% presented maculo-papular rash. A total of 82.73% cases reported contact with dogs parasitized by ticks, and in 75.23%, an eschar was present. IFA as a serologic test for confirmation was used in 38.8% of cases [44]. 

Although *Rickettsia slovaca* and *R. raoultii* are the most common etiological agents in SENLAT syndrome (i.e., *scalp eschar and neck lymphadenopathy after tick bite*), there are other germs responsible for it, such as Coxiella-like bacteria, while other bacteria such as *Rickettsia rioja*, *Rickettsia sibirica mongolitimonae*, *Rickettsia massiliae*, *Bartonella henselae*, *Coxiella burnetti*, *Borelia burgdorferi* and *Francisella tularensis* were isolated only sporadically [46]. These clinical aspects can also be found in children and women after Dermacentor tick bites. This syndrome is also known as DEBONEL (*Dermacentor-borne necrosis erythema and limphadenopathy*) [47]. This syndrome was described in a very rare situation in the Bucharest area but was never noticed in Constanta county. 

The case definition used for BF in Romania is based on clinical (i.e., fever, myalgia, maculo-papular rash, eschar), laboratory (i.e., immunofluorescence assay (IFA) with detection of increased antibody titer in pairs of sera) and epidemiological (i.e., recent tick bite, exposure to ticks in grass or contact with dogs) data [43].

## 5. Diagnosis

Often, the clinical and laboratory diagnosis of BF is challenging, and either the diagnosis is established very late or the diagnosis is not recognized, especially in non-endemic areas. In Romania, indirect IFA is used as a serologic diagnosis. Serological diagnosis via IFA requires serum collection in the acute phase of the disease and in convalescence; this second serum is usually difficult to collect as the patient is in convalescence. When only a single sample is collected, the interpretation can be challenging and must be correlated with the onset of symptoms. In the first week after the appearance of symptoms, when the patient presents to a physician for evaluation, the serology is usually negative. A high reactive titer with diagnostic significance appears in the second week of the disease, although a low or absent titer from the first week of the disease cannot completely exclude BF [26,27,28,29,30].

In recent years in Romania, a new serological method, enzyme-linked-immunoassay (ELISA), has been used for diagnosis. ELISA showed good sensitivity and specificity and proved its effectiveness in the diagnosis of rickettsial infections. It also has the advantage that a single blood sample is collected at the end of the first week of the disease [28,29,30]. The determination of immunoglobulin (Ig) M or Ig G antibodies by ELISA in the diagnosis of rickettsial infection is also used due to the reduced need for technical expertise. Although initially, ELISA tests were just qualitative tests, during the last few years, ELISA tests became more commonly used to unravel diagnostic information [30]. However, in Romania, ELISA is rarely used in daily medical practice for *R. conorii* detection.

The diagnosis of BF in the acute phase remains elusive under the conditions of the limits of serological and molecular tests [27,28,29]. In Romania, in 2010 at the Laboratory for Vector Borne Infections from National Institute Cantacuzino from Bucharest, the molecular diagnosis of BF was first established. All these cases were isolated in patients from five counties in southeastern Romania: Tulcea, Constanta, Prahova, Calarasi and Buzau. In Constanta and Tulcea counties in Romania, usually, the diagnosis is based on clinical aspects, and in the case of uncertainty, serum samples are tested using indirect IFA [48].

Other two molecular studies conducted by authors in tick populations from different regions of Romania identified four rickettsial pathogens: in Ixodes ricinus—*R. helvetica* and *R. monacensis*; and in *Dermacentor marginatus*—*R. slovaca* and *R. raoultii* [49,50].

In the Hospital of Infectious and Tropical Diseases, “Victor Babes” from Bucharest identified two cases of infection with *R. massiliae* and one case of each *R. slovaca* and *R. raoultii* between June 2011 and June 2012, with these being etiological agents of SENLAT syndrome [47,51].

When a rickettsial infection is considered and appropriate etiological treatment is administered, defervescence occurs in approximately 48 h and can also be considered a diagnostic test. The persistence of fever beyond this time interval occurs either in severe cases or when the correct diagnosis is not rapidly established [8,49,50,51].

## 6. Treatment

Before antimicrobial therapy, Brouqui and contributors [8] recommend that first, a blood sample should be collected early in the course of the disease, before starting the treatment, and a second sample should be obtained 2 weeks later. This study is supported by Figoni et al. [52] together with Stanek and Strle [53]. The authors also stated that the main reason to start the treatment in such patients is just to shorten the duration of the manifestation and to prevent the development of later complications. Although in Europe, the principle of “watch and wait” is still recommended [53], currently, in Romania, the treatment of BF in adults requires tetracycline (i.e., 500 mg/6 h, 7 days) or doxycycline (i.e., 200 mg/12 h, 7 days). In children, clarithromycin (i.e., 5 mg/kg every 12 h, 7 days) or azithromycin (i.e., 12 mg/kg/24 h, 5 days) can be used. Finally, in the case of pregnant women, josamycin is used (i.e., 3 g/day, 8 days) [54]. Chloramphenicol was used in the past; nowadays, it is no longer used [3].

In the Dobruja region, in the last ten years, prophylaxis with a dose of doxycicline (i.e., 200 mg) in adults and a dose of azitromycine (i.e., 12 mg/kgc) in children is recommended if the tick remained attached to the human body for more than 24 h [54]. 

## 7. Preventive Measures

People should be advised to avoid dogs parasitized by ticks and areas with grass and ticks during late spring, summer and early autumn. People living in endemic areas or people who travel in these endemic areas should perform routine tick checks. Also, people should wear long sleeves and light-colored clothes in order to notice ticks [22]. 

## 8. Conclusions

BF is still an endemic disease in southeastern Romania, and the trend towards an increase in severe cases is real. In any case with fever, rash and epidemiological data for vector-borne disease, BF should be suspected and the treatment should be immediately administered. Therefore, people living or traveling in endemic areas should perform routine tick checks as a preventive measure for BF detection and spread.

## Figures and Tables

**Table 1:** Symptoms and diagnosis of BF in Romania; results from different regions of the country.

Symptoms/Diagnosis	Authors		
	Cambrea [32]	Serban [33]	Pitigoi et al. [34]
Period	2006–2015	2000–2008	2000–2011
Area	Constanta	Romania	Bucharest
No of cases	533	786	339
Fever	97.5%	96%	99.4%
Headache	80.4%	56%	43.1%
Maculo-papular rash	97.18%	N/A *	98.2%
Eschar	75.23%	N/A *	57.9%
Contact with dog ticks	82.73%	96%	67.3%
Serology for confirmation	38.8%	38%	36.28%

* N/A = not available data.

## Data Availability

The data of this report are available from the corresponding authors upon request.
